# Prevalence of post-traumatic stress disorder, depression and somatisation in recently arrived refugees in Germany: an epidemiological study

**DOI:** 10.1017/S2045796019000325

**Published:** 2019-07-31

**Authors:** Y. Nesterko, D. Jäckle, M. Friedrich, L. Holzapfel, H. Glaesmer

**Affiliations:** Department of Medical Psychology and Medical Sociology, University of Leipzig, Leipzig, Germany

**Keywords:** Depression, epidemiology, mental health, multicultural, post-traumatic stress disorder

## Abstract

**Aims:**

Despite recent worldwide migratory movements, there are only a few studies available that report robust epidemiological data on the mental health in recent refugee populations. In the present study, post-traumatic stress disorder (PTSD), depression and somatisation were assessed using an epidemiological approach in refugees who have recently arrived in Germany from different countries.

**Methods:**

The study was conducted in a reception facility for asylum-seekers in Leipzig, Germany. A total of 1316 adult individuals arrived at the facility during the survey period (May 2017–June 2018), 569 of whom took part in the study (*N* = 67 pilot study and *N* = 502 study sample; response rate 43.2%). The questionnaire (11 different languages) included sociodemographic and flight-related questions as well as standardised instruments for assessing PTSD (PCL-5), depression (PHQ-9) and somatisation (SSS-8). Unweighted and weighted prevalence rates of PTSD, depression and somatisation were presented stratified by sex and age groups.

**Results:**

According to established cut-off scores, 49.7% of the respondents screened positive for at least one of the mental disorders investigated, with 31% suffering from somatisation, 21.7% from depression and 34.9% from PTSD; prevalence rates of major depression, other depressive syndromes and PTSD were calculated according to the DSM-5, which indicated rates of 10.3, 17.6 and 28.2%, respectively.

**Conclusions:**

The findings underline the dramatic mental health burden present among refugees and provide important information for health care planning. They also provide important information for health care systems and political authorities in receiving countries and strongly indicate the necessity of establishing early psychosocial support for refugees suffering from psychological distress.

## Introduction

According to the Office of the United Nations High Commissioner for Refugees (UNHCR), 68.5 million people were forcibly displaced worldwide by the end of 2017, of which 25.4 million have been acknowledged as refugees and 3.1 million as registered asylum-seekers (UNHCR, 2018). The number of forcibly displaced individuals has been growing for years – in many instances as a result of human rights violations due to armed conflicts and political instability in different parts of the world. Turkey hosted the largest number of refugees worldwide in 2017 (3.5 million), followed by Pakistan (1.4 million), Uganda (1.4 million), Lebanon (998.900), the Islamic Republic of Iran (979 400) and Germany (970 400) (UNHCR, 2018).

By now, there is striking evidence indicating that people who have to leave their homes because of armed conflicts, different kinds of organised violence, persecution and/or threats related to their ethnic, cultural or religious backgrounds, sexual orientation and/or political affiliations are exposed to a substantial level of psychological stressors, are more likely to be exposed to significant traumatic events and consequently are at a high risk of developing mental disorders (Fazel *et al*., 2005; Bogic *et al*., 2015; WHO, 2018). In general, research has focused on prevalence rates of and specific risk factors for common mental disorders, most often post-traumatic stress disorder (PTSD) and depression (Giacco *et al*., 2018). A closer look at the existing evidence on prevalence rates of mental disorders in refugee populations reveals a wide range of reported prevalence rates (e.g. 0–99% for PTSD and 3–85% for depression; Lindert *et al*., 2009; Steel *et al*., 2009). Moreover, only a few recent studies are available that report robust epidemiological data on the mental health of refugees specifically in light of recent worldwide migratory movements. For example, Steel *et al*. (2017) reported prevalence rates of 47% for PTSD and 20% for depression in refugees from predominantly sub-Saharan Africa (*N*  =  420) using stratified quota sampling based on Swedish census data. In a 2017 population-based survey by Tinghög *et al*., weighted prevalence rates of 40.2% for depression, 31.8% for anxiety and 29.9% for PTSD were reported in Syrian refugees (*N*  =  1215) who had resettled in Sweden. Cheung *et al*. (2018) used a convenience sampling method in their study, which found a PTSD prevalence rate of 43% among Syrian refugees (*N*  =  1197) in Sweden and Turkey and higher rates for those living in Turkey. Overall, existing findings, though ranging significantly between studies, consistently indicate significantly increased prevalence rates of mental disorders in refugees. This range may be attributable to (1) heterogeneity within the refugee populations (e.g. Syrian refugees *v.* refugees from Eritrea) in different host countries (e.g. Sweden *v.* Turkey) at different times of assessment (recently arrived refugees *v.* years after resettlement), (2) differences in methodology (e.g. sample size, sampling methods, selection bias) and (3) varying quality of instruments used (Giacco *et al*., 2018). In their recently published review on mental health among refugees, Giacco *et al*. (2018) conclude that studies of higher methodological quality based on more representative samples report lower prevalence rates compared to studies which used opportunistic or convenience samples. In addition, survey timing appears to be important when interpreting results, especially when considering (1) possible trauma exposure before, during and/or after flight, and (2) immigration-related risk factors reported by several studies as having long-term negative impacts on immigrants/refugees' mental health after resettlement (e.g. perceived discrimination, long asylum-application procedures, health care system barriers, restricted access to the labour market, etc.; Iversen and Morken, 2004; Johnson and Thompson, 2008; Bogic *et al*., 2012; Fazel *et al*., 2012). Despite the increasing number of refugees who have arrived in Europe since 2015, there is still a lack of robust epidemiological data on mental disorders, especially on symptoms of somatisation (Rohlof *et al*., 2014) in different refugee populations living in European countries in general, and especially in Germany, the host country with the largest population of recently arrived refugees in the European Union. Since somatisation is a common mental disorder in traumatised individuals and a common comorbidity of PTSD, it seems worthwhile to investigate symptoms of somatisation in refugees (Spitzer *et al*., 2008). Therefore, the aim of the present study is to use an epidemiological approach to report prevalence rates of PTSD, depression and somatisation in refugees who have recently arrived in Germany from different regions of origin.

## Methods

### Data collection and study sample

The study was conducted between May 2017 and June 2018 in a primary reception facility operated by the Federal State of Saxony for asylum-seekers in Leipzig, Germany. The study's target population^1^ consisted of the adult individuals (⩾18 years) who were currently being accommodated in the facility during the survey period. Based on the facility's registration data of all newly arrived residents, potential study participants were approached by members of the project staff in their accommodation unit, informed about the study objectives as well as data protection policy, and, in the event that they were willing to participate, introduced to the survey procedure. Between 1 and 15 May 2017, the participants were asked to fill out a paper version of the questionnaire (pilot study; *N*  =  67), after 17 May 2017, the participants filled out a tablet-based questionnaire in their native language. Correspondingly, information and recruitment procedures were carried out using language interpreters or study staff with appropriate language skills^2^. The following languages were available: Arabic, English, Farsi, French, German, Russian, Spanish, Tigrinya and/or Turkish. After information sheets and consent forms in the languages listed above were handed out and consent to participate was given, the participants responded to the questionnaire (time needed approximately: 45 min). Project staff was available to answer questions when necessary. The assessments took place three times a week, on Mondays, Wednesdays and Thursdays between 10 a.m. and 1 p.m. Recently arrived residents were approached first (arrival within the last 7 days) followed by residents who had arrived more than 7 days ago, but had yet to complete the questionnaire. Data were electronically transferred and administered consecutively to the ongoing data collection using LimeSurvey Offline-App for android systems. Data control and consistency checks were carried out at monthly intervals and a simple plausibility check was carried out immediately after the entry of a maximum of 30 data sets. Data were stored in an anonymous form on a computer at the University of Leipzig network in accordance with the data protection guidelines. No personal data were stored.

The study was approved by the Ethics Committee of the Medical Faculty of the University of Leipzig (446/16-ek). All study procedures were conducted in accordance with the Helsinki Declaration and its later amendments or comparable ethical standards. Written informed consent was obtained from all study participants.

### Instruments

The questionnaire used in the present study included sociodemographic and flight-related questions, as well as standardised instruments for assessing PTSD, and symptoms of depression and somatisation. The German version of the questionnaire was translated and back-translated into ten different languages (Albanian, Arabic, English, Farsi, French, Kurdish, Russian, Spanish, Turkish and Urdu) by a professional translation agency (mt-g medical translation GmbH) specialised in medical translations. The Tigrinya version of the questionnaire was translated and back-translated by the same agency based on the English version of the questionnaire. All back-translations were reviewed by the first and last authors and, when necessary, returned to the agency for final modification/adjustment. In the present study, the Albanian, Kurdish and Urdu versions of the questionnaires were not used due to the absence of native speakers of those languages among the study sample.

#### Sociodemographic and flight-related characteristics

Participants were asked to provide information about their age, sex, country of origin and/or country of birth, marital status, number of children, level of education, last occupation, duration of their flight, accompaniment during the flight, and current access to information about family members and friends who were left behind. In addition, length of stay in the facility was assessed using registration data provided by the facility staff.

#### Traumatic events

The occurrence of traumatic events was recorded using the revised DSM-5 Life Events Checklist (LEC-5) for assessing trauma exposure (Weathers *et al*., 2013). The LEC-5 is comprised of 16 items, which address the experience of 16 types of events that can potentially result in PTSD or distress. The following response categories are given for each type of event: (1) happened to me, (2) witnessed it, (3) learned about it, (4) part of my job, (5) not sure and (6) doesn't apply. The LEC-5 was used in combination with the PCL-5 for the purpose of establishing exposure to a PTSD A-Criterion, with the response option ‘happened to me’ (direct exposure) being the only one used in our study.

#### Post-traumatic stress disorder

PTSD was assessed with the PCL-5 (PTSD Checklist), a 20-item self-report instrument, which assesses symptoms of PTSD as defined by the DSM-5 (Blevins *et al*., 2015). The 20 items of the PCL-5 reflect the frequency with which respondents have experienced the item in question rated on a five-point Likert scale ranging from ‘not at all’ (0) to ‘extremely’ (4). A total score (0–80) can be obtained by summing up the scores for each of the 20 items. A score at or above the cut-off score of 33 indicates the presence of PTSD in the respondent. In addition, a provisional PTSD diagnosis may be determined by taking items that are rated 2 (‘moderately’) or higher into consideration following the PTSD diagnostic algorithm of the DSM-5: one B-item (items 1–5), one C-item (items 6–7), two D-items (items 8–14) and two E-items (items 15–20), with cluster B representing intrusion, cluster C avoidance, cluster D negative alterations in cognitions and mood and cluster E measuring alterations in arousal and reactivity. Cronbach's *α* in the present study was *α*  =  0.95 (0.93–0.97. for the different language versions).

#### Depression

Symptoms of depression were assessed with the Patient Health Questionnare-9 (PHQ-9; Kroenke *et al*., 2001). The PHQ-9 was used to assess both the severity of participants' depressive symptoms as well as aiding in making provisional diagnoses of major depressive disorder (MDD) and other depressive syndromes (ODS) based on the diagnostic criteria of the DSM-5. The PHQ-9 contains nine items rated on a scale of 0 (‘not at all’) to 3 (‘nearly every day’) which reflect the frequency with which participants have experienced the symptom in question within the previous 14 days. Based on the total sum (0–27), symptom severity can be divided into the categories ‘none–minimal’ (0–4), ‘mild’ (5–9), ‘moderate’ (10–14), ‘moderately severe’ (15–19) and ‘severe’ (20–27) depression. Participants with a sum score of >14 were classified as having a depressive disorder. An MDD was diagnosed if the obligatory symptoms (items 1 and 2) were present AND the respondent experienced five or more additional items at least ‘more than half of the days’ (2). A diagnosis of ODS was determined if items 1 and 2 screened positively AND the respondent experienced two, three or four additional items ‘more than half of the day’. Cronbach's *α* in the present study was *α*  =  0.84 (0.70–0.89 for the different language versions).

#### Somatisation

Somatic symptoms were assessed with the Somatic Symptom Scale-8 (SSS-8), a brief self-reported measure of somatic symptom burden (Gierk *et al*., 2014). The SSS-8 is a shortened version of the PHQ-15 questionnaire developed for DSM-5 field trials. Each item can be rated on a five-point Likert scale from ‘not at all’ (0) to ‘very much’ (4) referring to the previous 7 days. The total scores therefore range from 0 to 32, and are subdivided into five categories of severity: ‘none to minimal’ (0–3), ‘low’ (4–7), ‘medium’ (8–11), ‘high’ (12–15) and ‘very high’ (16–32) somatic symptom burden. A cut-off score of >11 was used for the present study. Participants with a sum score of >11 were diagnosed with somatisation. The internal consistency was *α*  =  0.84 (0.77–0.93 for the different language versions).

### Statistical analyses

Statistical analyses were performed using the IBM SPSS statistical package, version 24.0 for Windows. Descriptive statistics and *χ*^2^-tests were used to test possible selection bias between the entire population of newly arrived refugees in the facility and the study sample. Prevalence rates were calculated according to the cut-off scores or algorithms of each questionnaire. In the present study, unweighted and weighted prevalence rates (weighted by country of origin) of PTSD, depression and somatisation were presented in total, stratified by sex and age groups and for the different points of assessment based on how recently the participants had arrived (e.g. within the past 7 days).

## Results

### Sample characteristics and procedure

**Fig. 1.**
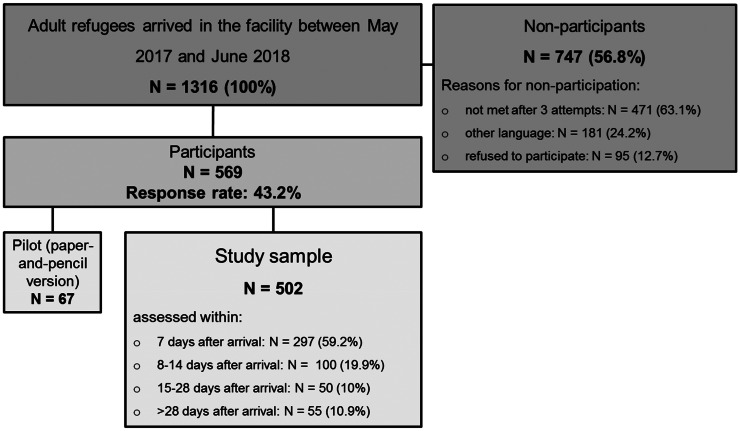
Study procedure.

A total of 1316 adult individuals were newly accommodated in the primary reception facility during the survey period, 569 of whom took part in the study. Of these, 67 individuals filled out the paper version of the questionnaire (pilot study) and 502 (study sample) responded via tablet (response rate 43.2%). Figure 1 gives an overview of the study procedure.

A total of 297 (59.2%) participants were assessed within 7 days after their arrival at the facility, 100 individuals (19.9%) between 8 and 14 days, 50 participants (10%) between 15 and 28 days and another 55 individuals (10.9%) participated more than 28 days after their arrival. The majority of non-participants (63.1%; *n*  =  417) were residents who could not be contacted after three attempts to visit them (not present at the allocated accommodation unit), 24.2% (*n*  =  181) could not be included due to the fact that the questionnaire was not available in their native language, and 95 individuals (12.7%) refused participation. Data on all non-participants' age, sex and country of origin were recorded to identify possible selection bias.

### Sociodemographic and flight-related characteristics

Table 1 gives an overview of the study sample's sociodemographic and flight-related characteristics as well as the distribution of age, sex and country of origin among participants and non-participants. There were no significant differences between participants and non-participants with respect to age (*χ*^2^(3, 1313)  =  3.32, *p*  =  0.344) and sex (*χ*^2^(1, 1316)  =  0.44, *p*  =  0.506). A significant difference between participants and non-participants was found regarding their countries of origin (*χ*^2^(8, 1313)  =  172.83, *p* < 0.001), indicating a mild selection bias for the country of origin.

**Table 1.** Sociodemographic and flight-related characteristics

	Participants *N* = 502	Non-participants *N* = 814^1^	*χ*^2^	Total *N* = 1316
Age				
*M*/s.d./range	29.73/8.79/18–70	29.48/10.5/18–90^2^		29.55/9.88/18–90^3^
18–29 years	293 (58.3%)	504 (62.1%)	3.324	797 (60.7%)
30–39 years	142 (28.3%)	200 (24.6%)		342 (26%)
40–49 years	44 (8.8%)	62 (7.6%)		106 (8.1%)
>50 years	23 (4.6%)	46 (5.7%)		69 (5.2%)
Sex				
Male	348 (69.3%)	550 (67.6%)	0.441	898 (68.2%)
Female	154 (30.6%)	264 (32.4%)		418 (31.8%)
Country of origin				
Cameroon	92 (18.3%)	60 (7.4%)	172.832*	152 (11.6%)
Eritrea	41 (8.2%)	171 (21%)		212 (16.1%)
Iraq	23 (4.6%)	40 (4.9%)		63 (4.8%)
Nigeria	38 (7.6%)	36 (4.4%)		74 (5.6%)
Syria	52 (10.4%)	69 (8.5%)		121 (9.2%)
Turkey	43 (8.6%)	44 (5.4%)		87 (6.6%)
Venezuela	85 (16.9%)	28 (3.4%)		113 (8.6%)
Other^a^	128 (25.4%)	366 (45%)		494 (37.5%)
Years of schooling				
*M*/s.d./range	10.6/3.78/0–15			
University degree^4^				
Yes	259 (51.9%)			
No	240 (48.1%)			
Last occupational status^5^				
Employed	107 (21.4%)			
In retirement	4 (0.8%)			
Military service	23 (4.6%)			
Self-employed	121 (24.2%)			
Studies or training	108 (21.6%)			
No employment	61 (12.2%)			
Other	77 (15.4%)			
Marital status^5^				
Single	290 (57.9%)			
Married	180 (35.9%)			
Divorced	21 (4.2%)			
Widowed	10 (2%)			
Partnership^5^				
Yes	186 (37.1%)			
No	315 (62.9%)			
Children^5^				
Yes	201 (40.1%)			
No	300 (59.9%)			
Flight duration in month				
*M*/s.d./range	7.4/11.29/0–156			
Accompaniment during the flight^5^				
Alone	224 (44.7%)			
Strangers	127 (25.3%)			
Friends	50 (10%)			
Family members	100 (20%)			
Information about family^5^				
Yes	266 (53.1%)			
No	235 (46.9%)			
Information about friends^4^				
Yes	186 (37.3%)			
No	313 (62.7%)			

*p < 0.001; ^1^including *N*  =  67 pilot study; ^2^*N*  =  812; ^3^*N*  =  1314; ^4^*N*  =  499; ^5^*N*  =  501.

a Country of origin other (*N*, total sample): Afghanistan (25), Albania (6), Algeria (9), Armenia (14), Azerbaijan (1), Belarus (1), Colombia (1), Côte d'Ivoire (1), Czech Republic (1), Ethiopia (50), Gambia (3), Ghana (6), Georgia (76), Guinea (3), India (19), Iran (10), Jordan (2), Kosovo (2), Kuwait (2), Lebanon (14), Libya (45), Macedonia (2), Morocco (9), Mozambique (1), Myanmar (12), Niger (1), Palestine (19), Pakistan (25), Russian Federation (28), Senegal (2), Serbia (6), Somalia (52), Sri Lanka (1), Tajikistan (1), Togo (1), Tunisia (18), Ukraine (1), Vietnam (4), Yemen (2), stateless (16).

The mean age of the participants in the present study was 29.73 (s.d.  =  8.79) years. The majority of the participants were male (*n*  =  348, 69.3%). The largest groups were participants from Cameroon (18.3%), Venezuela (16.9%) and Syria (10.4%); all in all, participants from over 30 different countries took part in the survey. The average number of years of schooling the participants had received was 10.6 (s.d.  =  3.78), and 51.9% of the study sample reported having a university degree. A total of 290 (57.9%) participants were single, 35.9% (*n*  =  180) were married, 4.2% (*n*  =  21) divorced and 2% (*n*  =  10) widowed, with 186 (37.1%) participants reporting that they have a partner and 201 (40.1%) that they have children. The mean flight duration was 7.4 months (s.d.  =  11.29), reflecting a wide range spanning from 0 to 156 months, with 44.7% (*n*  =  224) of the participants reporting that they had been alone while fleeing. A total of 235 (46.9%) participants reported that they currently have no access to information about their family members, and 313 (62.7%) had no information about friends they had left behind.

### Traumatic events

**Fig. 2.**
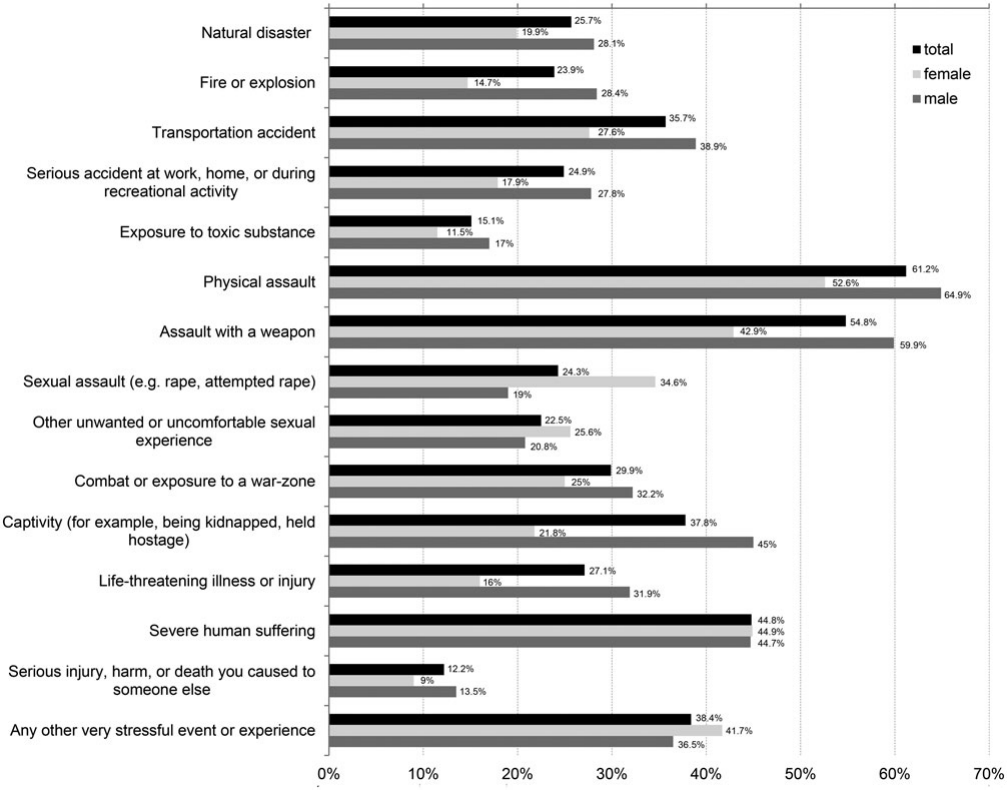
Prevalence of traumatic events.

The mean number of traumatic events this study's participants reported having experienced was 4.78 (s.d.  =  3.75), with an overall range of 0–15 events. In Fig. 2, frequencies of the different traumatic events assessed in the present study are displayed for the entire sample stratified by sex. With rates of 61.6% (*n*  =  307) in total, 52.6% (*n*  =  82) in females and 64.4% (*n*  =  222) in males, physical assault was the event most frequently reported, followed by assault with a weapon (54.8% (*n*  =  275) in total, 42.9% (*n*  =  67) of female and 59.9% (*n*  =  205) of male participants), severe human suffering (44.8% (*n*  =  225) in total, 44.9% (*n*  =  70) of female and 44.7% (*n*  =  153) of male participants) and captivity (37.8% (*n*  =  190) in total, 21.8% (*n*  =  34) of female and 45% (*n*  =  154) of male participants). Sexual assaults were reported by 24.3% (*n*  =  152) of the participants (34.6% (*n*  =  54) of females and 19% (*n*  =  65) males). In general, 75.7% (*n*  =  380) of participants were exposed to interpersonal traumatic events, and 85.5% (*n*  =  426) reported having experienced at least one traumatic event.

### Prevalence rates of somatisation, depression and post-traumatic stress disorder

Due to differences in the distribution of country of origin between participants and non-participants (see Table 1), non-response weights by country of origin were applied when calculating the prevalence rates of somatisation, depression and PTSD. In Table 2, weighted and unweighted prevalence rates are reported for the entire sample as well as for the subsample assessed within the first 7 days, in each case stratified by sex. Overall, there were no substantial differences between weighted and unweighted prevalence rates and minimal differences due to the time of assessment. Using established cut-off scores for the different instruments, weighted prevalence rates of 31% were calculated for somatisation (44.1% in female and 23.9% in male participants; *χ*^2^(11 122)  =  39.14, *p* *<* 0.01), 21.7% for depression (22.1% in female and 21.5% in male participants) and 34.9% for PTSD (37.2% for females and 33.9% for males). Using diagnostic algorithms according to the DSM-5, major depression was identified in 10.3% (9.4% in females and 10.4% in males), ODS in 17.6% (19.5% in females and 16.8% in males) and PTSD in 28.2% of the study sample with higher rates observed among female participants (34 *v.* 25.6%; *χ*^2^(11 126)  =  8.32, *p* *<* 0.01).

**Table 2.** Prevalence rates of somatisation, depression and post-traumatic stress disorder by gender and assessment time: weighted and unweighted data

	Weighted by country of origin								Unweighted							
	Assessed within first 7 days				Total sample				Assessed within first 7 days				Total sample			
	Female (%)	Male (%)	*χ*^*2*^	Total (%)	Female (%)	Male (%)	*χ*^*2*^	Total (%)	Female	Male	*χ*^*2*^	Total	Female	Male	*χ*^*2*^	Total
Somatisation																
SSS-8 cut-off > 11	46.3	21.4	39.97**	29	44.1	25.3	39.14**	31	48.9% *N* = 90	21.3% *N* = 207	22.97**	29.6% *N* = 297	46.1% *N* = 154	24.8% *N* = 347	22.53**	31.3% *N* = 501
Depression																
MDD	10.5	9.9	0.06	10.1	9.4	10.7	0.43	10.3	12.2% *N* = 90	10.1% *N* = 207	0.28	10.8% *N* = 297	11.8% *N* = 153	10.1% *N* = 347	0.32	10.6% *N* = 500
ODS	21.6	14.5	4.80*	16.6	19.5	16.8	1.20	17.6	17.8% *N* = 90	13% *N* = 207	1.13	14.5% *N* = 297	18.3% *N* = 153	16.4% *N* = 347	.26	17% *N* = 500
PHQ-9 cut-off > 15	23.2	22.1	0.09	22.4	22.1	21.5	0.06	21.7	24.4% *N* = 90	20.3% *N* = 207	0.64	21.5% *N* = 297	24.2% *N* = 153	20.5% *N* = 347	0.87	21.6% *N* = 500
PTSD																
PTSD according to DSM-5	36.8	23.7	11.45**	27.7	34	25.6	8.32**	28.2	36.7% *N* = 90	25.1% *N* = 207	4.09*	28.6% *N* = 297	35.1% *N* = 149	25.6% *N* = 347	4.72*	28.5% *N* = 502
PCL-5 cut-off > 33	39.8	30.3	5.27*	33.2	37.2	33.9	1.06	34.9	39.8% *N* = 88	30.1% *N* = 206	2.61	33% *N* = 294	38.9% *N* = 154	32.9% *N* = 348	1.70	34.7% *N* = 496

*p < 0.05; **p < 0.01.

In addition, weighted and unweighted prevalence rates for somatisation, depression and PTSD were calculated stratified by sex and age groups (18–30 *v.* >30 years). The results are shown in Table 3. All in all, with the exception of somatisation rates (28.7 *v.* 34.9%; *χ*^2^(11 123)  =  4.77, *p* *<* 0.05), no differences were found between the age groups 18–30 and >30 years.

**Table 3.** Prevalence rates of somatisation, depression and post-traumatic stress disorder by gender and age group: weighted and unweighted data

	Weighted by country of origin							Unweighted						
	Female		Male		Total			Female		Male		Total		
	18–30 years (%)	>30 years (%)	18–30 years (%)	>30 years (%)	18–30 years (%)	>30 years (%)	*χ*^*2*^	18–30 years	>30 years	18–30 years	>30 years	18–30 years	>30 years	*χ*^*2*^
Somatisation														
SSS-8 cut-off > 11	39.9	49.3	24.4	27.1	28.7	34.9	4.77*	39.8% *N* = 83	53.5% *N* = 71	25.5% *N* = 208	23.7% *N* = 139	29.6% *N* = 291	33.8% *N* = 210	1.03
Depression														
MDD	6.4	12.7	10.8	10.9	9.6	11.5	1.05	8.4% *N* = 83	15.7% *N* = 70	10.6% *N* = 208	9.4% *N* = 139	10% *N* = 291	11.5% *N* = 209	0.29
ODS	19.7	19.2	18.2	14.1	18.5	15.9	1.26	21.7% *N* = 83	14.3% *N* = 70	18.3% *N* = 208	13.7% *N* = 139	19.2% *N* = 291	13.9% *N* = 209	2.48
PHQ-9 cut-off > 15	22.9	21.2	18.7	26.4	19.8	24.7	3.70	26.5% *N* = 83	21.4% *N* = 70	19.7% *N* = 208	21.6% *N* = 139	21.6% *N* = 291	21.5% *N* = 209	0.001
PTSD														
PTSD according to DSM-5	30.9	38.2	26.3	24.4	27.6	29.2	0.36	31.3% *N* = 83	39.4% *N* = 71	27.9% *N* = 208	22.1% *N* = 140	28.9% *N* = 288	28% *N* = 208	0.05
PCL-5 cut-off > 33	32.2	42.2	33.7	34.3	33.7	36.8	1.09	34.6% *N* = 81	44.1% *N* = 68	33.8% *N* = 207	31.4% *N* = 140	34% *N* = 291	35.6% *N* = 211	0.13

*p < 0.05.

**Fig. 3.**
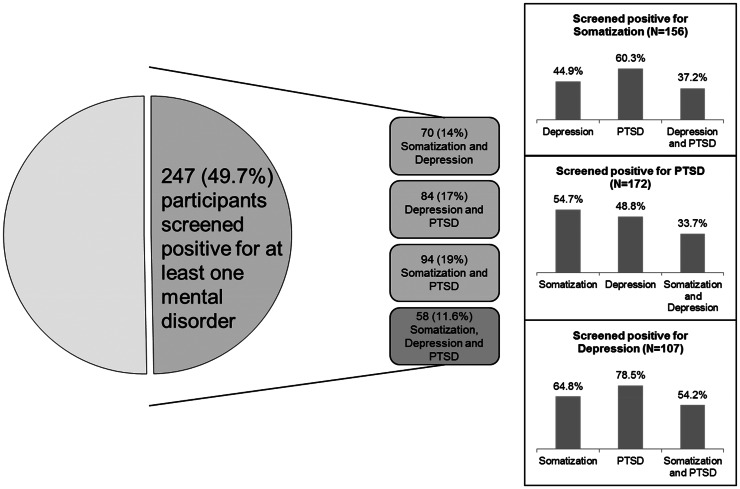
Comorbidity patterns of PTSD, depression and somatisation in the study sample (*N*  =  497).

Finally, 49.7% of the study sample (59.7% female and 45.4% male participants) screened positive for at least one of the mental disorders assessed in the study according to the cut-off scores for the SSS-8, PHQ-9 and PCL-5. The comorbidity patterns of somatisation, depression and PTSD in all possible combinations are shown in Fig. 3.

## Discussion

In the present study, prevalence rates for PTSD, depression and somatisation were assessed in refugees who had recently arrived in Germany. Due to detected differences between participants and non-participants with respect to the country of origin, both unweighted and weighted prevalence rates were presented using an epidemiological approach. In total, clinically relevant somatisation was found in 31%, depression in 21.7% and PTSD in 34.9% of the participants. According to the established cut-off scores of the instruments used in the present study, about half of the respondents (49.7%) screened positive for at least one of the mental disorders we investigated. In addition, prevalence rates of major depression, ODS and PTSD as defined by the DSM-5 were found to be 10.3% (major depression), 17.6% (ODS) and 28.2% (PTSD). At this point, the findings are in line with previous research that has observed very high prevalence rates of PTSD and depression among various refugee populations (Steel *et al*., 2017; Tinghög *et al*., 2017), despite the fact that the rates detected in the present study tended to be somewhat lower (e.g. 47% of PTSD in Steel *et al*. (2017) or 40.2% of depression in Tinghög *et al*. (2017)). Looking at the sociodemographic characteristics of the sample, e.g. relatively high level of education reported by the participants, further analyses are needed that address possible differences to the general refugee population in Germany. However, to the best of our knowledge, only a few studies are currently available having used a similar methodology as the present study, a limit which makes it difficult to compare our findings with those of others (Giacco *et al*., 2018). Moreover, no conclusive statement can be made about somatisation since, to the best of our knowledge, no previous studies have reported on that. When interpreting findings and drawing conclusions, special attention should be paid to the detected comorbidity patterns; in numerous studies, the high comorbidity between PTSD and depression in refugees has been reported as well as some findings which indicate higher levels of chronic pain resulting from psychological distress and/or trauma (Rohlof *et al*., 2014; Giacco *et al*., 2018). In light of this, overlapping symptoms and comorbid mental disorders caused by significant traumatic events experienced before and/or during flight seem likely; 85.5% of participants were exposed to at least one traumatic event, and 75.7% were exposed to interpersonal traumatic events, factors known to increase the probability of a mental disorder developing.

Although the present study has some major strengths – (1) epidemiological approach with available information about the entire population and basic characteristics of the non-responders to test for possible selection bias, (2) assessment of recently arrived refugees, considering the time frame of symptom burden (previous 7 days, previous 14 days, previous 4 weeks) leading to a rather homogenous sample with respect to exposure to post-migratory risk factors, (3) application of standardised instruments being translated and back-translated into 11 different languages, enabling refugees from over 30 different countries to take part in the study, and (4) assessment of somatisation, something which has not been systematically investigated in a comparable population before – there are some factors that limit how our results can be interpreted and consequently illuminate some important implications for future research. First, the prevalence rates detected in the present study are based on a cross-sectional investigation. In the future, longitudinal studies should be conducted to investigate and understand the trajectories of trauma-related mental health outcomes in refugees (e.g. remission rates and chronicity as well as the associated risk and protective factors). Although our study provides robust information about the prevalence of the most common mental disorders in recently arrived refugees, longitudinal investigations could inform future prevention and intervention strategies more precisely. In addition, the present study focuses on PTSD, depression and somatisation only, although increased prevalence rates of other mental disorders (e.g. anxiety disorders, substance use disorders or schizophrenia and other psychotic disorders) have been reported in different studies in refugee populations (Giacco *et al*., 2018). Second, due to the heterogeneity of refugee populations, both with regard to their countries of origin as well as with regard to the receiving countries and the conditions provided there, international collaborative research is needed in the future. So far, epidemiologically robust research that has been done on mental health in refugees has been conducted focusing almost solely on refugees residing in Western countries. The vast majority of refugees worldwide however are located either within their countries of origin or in bordering regions. In light of this fact, the generalisability of the detected prevalence rates is compromised, despite the methodological strengths of the present study. Considering the sociodemographic and flight-related characteristics of the study sample, the findings provide prevalence rates on a certain wave of refugees that reflect political crises and/or armed conflicts in specific countries (e.g. subgroups from Cameroon, Syria or Venezuela) on the one hand and possible selection bias due to the federal regulation on allocation of refugees within Germany on the other hand. Third, as no assessment of medical physical health was made, the detected rate of somatisation may be somehow affected. Finally, there is a general issue of measurement invariance, meaning the equivalence of a measured construct across different cultures, ethnic groups and/or language versions of instruments. The debate about cross-cultural research has long focused on the importance of functional equivalence or the comparability of validity coefficients or optimal cut-off scores Chen (2008). The development of measurement invariance tests and advanced statistical tools has however instigated more rigorous tests of measurement invariance (Dere *et al*., 2015). As a result, future research should focus on differences in mental health outcomes based on different cultural settings so that a robust frame for development and evaluation of culturally sensitive instruments can be built.

Despite the limitations and the implications for future research discussed and mentioned above, the results of the present study strongly suggest an urgent need to provide refugees with early professional psychosocial support and/or specific intervention programmes. Especially due to several barriers (e.g. language difficulties, lack of interpreters, financial regulations and access to care during the asylum procedure), the mental health care currently provided to refugees in Germany is insufficient (Bozorgmehr *et al*., 2016). Of an estimated 379 848 refugees in need of mental health care in 2015, only about 5% received some treatment in Germany (BafF, 2016). Untreated mental health problems can lead to a chronic course resulting in social withdrawal and lack of ability to integrate. Besides the associated problems for those individuals suffering from mental disorders and their families, restricted access to mental health care can also be associated with increased costs of care (Bozorgmehr and Razum, 2015). In light of this, the results of the present study underline the need for early assessment/screening of common mental disorders in refugees due to (1) possible reduction of mental symptoms at an early stage, (2) inform provision of health care for subgroups of urgent need, (3) support access to specialised mental health care and thus (4) reducing the risk of chronic trajectories of mental disorders. Key players in health care systems and among the political authorities of receiving countries need to be aware of the results presented here and their strong implications concerning the necessity of establishing early intervention programmes for refugees suffering from psychological distress. From our point of view, high-income Western countries currently have a specific responsibility to fulfil their humanitarian obligations by ensuring appropriate health care for the refugees they host – the sooner the better.

## Availability of data and materials

The data will not be shared due to ongoing analyses.

## Footnotes

1 Usually refugees arriving in the facility apply for asylum during the first 2 weeks after arrival. Thus formally all participants of the present study will become asylum-seekers, but due to the date of participation some of them were not asylum-seekers yet. In light of this, we prefer to use the term ‘refugees’ that seems to describe the participants of the study properly.

2 All interpreters in the project were informed about study procedure, ethical obligations, and have received training focusing on culture-sensitive diagnostics.
